# Virulence Characteristics of Biofilm-Forming *Acinetobacter baumannii* in Clinical Isolates Using a *Galleria mellonella* Model

**DOI:** 10.3390/microorganisms9112365

**Published:** 2021-11-16

**Authors:** Mahmoud A. F. Khalil, Fatma A. Ahmed, Ahmed F. Elkhateeb, Eman E. Mahmoud, Mona I. Ahmed, Randa I. Ahmed, Amal Hosni, Saad Alghamdi, Ahmed Kabrah, Anas S. Dablool, Helal F. Hetta, Sawsan S. Moawad, Enas Mamdouh Hefzy

**Affiliations:** 1 Department of Microbiology and Immunology, Faculty of Pharmacy, Fayoum University, Fayoum 63514, Egypt; 2 Department of Medical Microbiology and Immunology, Faculty of Medicine, Fayoum University, Fayoum 63514, Egypt; fan11@fayoum.edu.eg (F.A.A.); emh01@fayoum.edu.eg (E.M.H.); 3 Department of Critical Care Medicine, Faculty of Medicine, Fayoum University, Fayoum 63514, Egypt; Afm02@fayoum.edu.eg; 4 Department of Clinical and Chemical Pathology, Faculty of Medicine, Fayoum University, Fayoum 63514, Egypt; Esm02@fayoum.edu.eg; 5 Department of Chest Diseases and Tuberculosis, Faculty of Medicine, Fayoum University, Fayoum 63514, Egypt; Mia05@fayoum.edu.eg; 6 Department of Chest Diseases, Faculty of Medicine, Fayoum University, Fayoum 63514, Egypt; ria00@fayoum.edu.eg; 7 Department of Clinical Pathology, Faculty of Medicine, Assiut University, Assiut 71515, Egypt; amal.alameldin@yahoo.com; 8 Laboratory Medicine Department, Faculty of Applied Medical Sciences, Umm Al-Qura University, Makkah 24381, Saudi Arabia; ssalghamdi@uqu.edu.sa (S.A.); amkabrah@uqu.edu.sa (A.K.); 9 Department of Public Health, Health Sciences College at Al-Leith, Umm Al-Qura University, Makkah 24381, Saudi Arabia; Asdablool@uqu.edu.sa; 10 Department of Medical Microbiology and Immunology, Faculty of Medicine, Assiut University, Assiut 71515, Egypt; 11 National Research Center (N.R.C), Department of Pests and Plant protection, Giza 12311, Egypt; abzs9999@yahoo.com

**Keywords:** *Acinetobacter baumannii*, pneumonia, biofilm, carbapenem, *Galleria mellonella*

## Abstract

*Acinetobacter baumannii* is a Gram-negative coccobacillus responsible for severe hospital-acquired infections, particularly in intensive care units (ICUs). The current study was designed to characterize the virulence traits of biofilm-forming carbapenem-resistant *A. baumannii* causing pneumonia in ICU patients using a *Galleria mellonella* model. Two hundred and thirty patients with hospital-acquired or ventilator-associated pneumonia were included in our study. Among the total isolates, *A. baumannii* was the most frequently isolated etiological agent in ICU patients with pneumonia (54/165, 32.7%). All *A. baumannii* isolates were subjected to antimicrobial susceptibility testing by the Kirby–Bauer disk diffusion method, while the minimum inhibitory concentrations of imipenem and colistin were estimated using the broth microdilution technique. The biofilm formation activity of the isolates was tested using the microtiter plate technique. Biofilm quantification showed that 61.1% (33/54) of the isolates were strong biofilm producers, while 27.7% (15/54) and 11.1% (6/54) showed moderate or weak biofilm production. By studying the prevalence of carbapenemases-encoding genes among isolates, *bla*_OXA-23_-_like_ was positive in 88.9% of the isolates (48/54). The *Bla_NDM_* gene was found in 27.7% of the isolates (15/54 isolates). *Bla*_OXA-23_-_like_ and *bla_NDM_* genes coexisted in 25.9% (14/54 isolates). *Bap* and *bla*_*PER*-*1*_ genes, the biofilm-associated genes, coexisted in 5.6% (3/54) of the isolates. For in vivo assessment of *A. baumannii* pathogenicity, a *Galleria mellonella* survival assay was used. *G. mellonella* survival was statistically different between moderate and poor biofilm producers (*p* < 0.0001). The killing effect of the strong biofilm-producing group was significantly higher than that of the moderate and poor biofilm producers (*p* < 0.0001 for each comparison). These findings highlight the role of biofilm formation as a powerful virulence factor for carbapenem-resistant *A. baumannii* that causes pneumonia in the ICU.

## 1. Introduction

Pneumonia and other lower respiratory tract infections (LRTIs) affect about 10–25% of patients in the intensive care unit (ICU), resulting in approximately 22–71% mortality among these patients [1,2]. The causative infectious agent cannot be discriminated clinically, and their incidence have a variable geographic prevalence [3,4]. *Acinetobacter baumannii* is a Gram-negative coccobacillus responsible for severe hospital-acquired infections, particularly in the ICU. It causes infections such as bacteremia, pneumonia, urinary tract infection, meningitis, and wound infection [5]. The mortality rate among ICU patients infected by *A. baumannii* is 45–60%, which may increase to over 80% when extensive drug resistance (XDR) among these pathogens is involved [6]. Moreover, insufficient therapeutic options are available for infections caused by multiple drug-resistant (MDR) *A. baumannii* strains [7,8].

A major obstacle in controlling *A. baumannii* infections is their ability to survive on abiotic surfaces, such as catheters and endotracheal tubes, which makes them a critical source of nosocomial infections [9]. Biofilm formation ability is believed to be the leading cause of *A. baumannii* persistence in harsh environments [9,10], and an important virulence trait of *A. baumannii*. In addition, considerable studies have highlighted the role of biofilms in the protection of *A. baumannii* against host immune defense [11,12]. Consequently, biofilm-forming strains can induce troublesome infections.

Biofilms consist mainly of polysaccharides, proteins, and extracellular DNA. Biofilm formation is controlled by various factors, including environmental signals, sensing molecules, cation concentrations, and many genetic determinants [13]. Biofilm-associated proteins (Bap) are large multi-domain proteins that play an essential role in cell adhesion and biofilm formation in both Gram-positive and Gram-negative bacteria. Inactivation of such proteins can result in reduced biofilm development capacity [14,15]. Moreover, a study carried out by Sechi et al. indicated that *bla*_*PER*-*1*_ (extended-spectrum β-lactamase PER-1) expression was associated with enhanced surface adhesion [16].

In addition, outer membrane protein A (OmpA) is an important protein that mediates antibiotic resistance, biofilm formation, immunomodulation, and eukaryotic cell infection [6]. The overproduction of the outer membrane protein A is associated with a high risk of mortality due to hospital-acquired pneumonia and bacteremia associated with *A. baumannii* [17]. In addition, the level of expression of *ompA* can be considered a quick diagnostic for antibiotic resistance in *A. baumannii*. Traditional minimum inhibitory concentration (MIC) analyses are consistent with the level of expression of *ompA* [18].

The current study was designed to evaluate the role of carbapenem-resistant *A. baumannii* (CRAB) in causing pneumonia in ICU patients. An investigation into the biofilm formation ability of these clinically significant isolates was carried out, as well as its biofilm-related genes, *ompA*, *bap*, and *bla*_*PER*-*1*_. *Galleria mellonella,* a caterpillar of the greater wax moth, is a comparatively simple, non-mammalian model that can be used to study host-pathogen interactions in vivo [19,20]. This model was used for the in vivo evaluation of the virulence traits of *A. baumannii* isolates related to their biofilm-forming capacities.

## 2. Methods

### 2.1. Study Settings

The present study was conducted at the Department of Medical Microbiology and Immunology, Faculty of Medicine, Fayoum University, Fayoum, Egypt. Patients were recruited from two ICUs, the General Intensive Care Unit (GICU) and the Cardiac Intensive Care Unit (CICU), in the period from May 2019 to February 2021, at the Fayoum University Hospital. The study was affirmed by the Research Ethics Committee at the Faculty of Medicine, Fayoum University (Code: R92). Patients with hospital-acquired pneumonia (HAP) and ventilator-associated pneumonia (VAP) were recruited for this study.

Diagnosis of HAP and VAP were based on guidelines provided by the American Thoracic Society/Infectious Disease Society of America (ATS/IDSA). Briefly, clinically suspected cases had a radiographic and microbiologic examination and, to obtain diagnostic information and specimens for culture sensitivity to direct therapy, bronchoscopy was used if available [21].

### 2.2. Specimen Collection and Processing and Bacterial Isolates Identification

All collected respiratory samples, including sputum, bronchoalveolar lavage (BAL), endotracheal aspirates (ETA), or pleural fluid from ICU patients with pneumonia were considered for analysis. The samples were transported to the microbiology laboratory and processed without any delay. The clinical samples were cultured on MacConkey agar and 5% sheep blood agar (Oxoid, Basingstoke, UK) and incubated overnight at 37 °C. A Gram-stained smear was made from all specimens and examined under a light microscope. Sputum samples with less than 10 squamous epithelial cells or more than 25 pus cells/low-power field were considered for culture analysis [22].

Isolate identification was accomplished by conventional microbiological methods [23] and the API 20NE system (bioMérieux, Marcy l’Etoile, France) [24]. Identification was confirmed with the VITEK 2 system according to the manufacturer’s recommendations. The confirmed *A. baumannii* isolates were stored at −80 °C until further processing. Isolates were serially encoded from A701 to A754. Furthermore, isolates identity was affirmed by species-specific PCR for the *bla_OXA-51-like_* gene [25].

### 2.3. The Antimicrobial Susceptibility Testing

#### 2.3.1. Kirby–Bauer Disk Diffusion Method

All *A. baumannii* isolates from ICU patients with pneumonia were subjected to antimicrobial susceptibility testing by the Kirby–Bauer disk diffusion method according to the instructions of the Clinical and Laboratory Standards Institute (CLSI) recommendations [26]. The following antibiotics were tested: ampicillin–sulbactam (SAM, 10/10 μg), ceftazidime (CAZ, 30 μg), imipenem (IMP, 10 μg), amikacin (AK, 30 μg), ciprofloxacin (CIP, 5 μg), doxycycline (DO, 30 μg), gentamicin (CN, 10 μg), and colistin (CO, 10 μg). *Pseudomonas aeruginosa* ATCC 27,853 and *Escherichia coli* ATCC 25,922 were used as controls.

#### 2.3.2. Minimal Inhibitory Concentration

The minimum inhibitory concentrations (MICs) of the antibiotics imipenem and colistin) were estimated using the broth microdilution technique [26]. *Pseudomonas aeruginosa* ATCC 27,853 and *Escherichia coli* ATCC 25,922 were used as controls. For colistin MIC determination, serial dilutions of colistin ranging from 0.25 to 32 μg/mL were used. Resistant and susceptible to colistin were defined as MIC ≥ 4 μg/mL and ≤2 μg/mL, respectively [26]. *Escherichia coli* NCTC13846 (mcr + ve), *E. coli* ATCC 25922, and *P. aeruginosa* ATCC 27,853 were used as controls for colistin MIC testing.

### 2.4. Phenotypic Detection of Carbapenemases by Triton Hodge Test (THT)

Using sterile swabs, approximately 50 µL of pure Triton X-100 (Sigma-Aldrich, St. Louis, MO, USA) was poured onto the center of a Mueller-Hinton agar (MHA) plate and immediately coated across the entire plate in 4 to 6 directions. For around 10 min, the plate was left undisturbed until the reagent was entirely absorbed. THT’s subsequent procedures were identical to those used for Modified Hodge test (MHT) [27]. The specific technique of MHT was performed as previously reported [28]. The test was carried out using meropenem disc (10 μg). *K. pneumoniae* ATCC BAA-1705 and ATCC BAA-1706 strains were used as positive and negative controls respectively.

### 2.5. Biofilm Formation Test

The biofilm formation activity of *A. baumannii* isolates was tested using the microtiter plate technique. Isolates were cultured overnight in Tryptic soy broth (TSB) at 37 °C. (Oxoid, Basingstoke, UK) and adjusted to 0.5 McFarland standards. Twenty microliters of fresh bacterial culture was inoculated in 180 µL of TSB supplemented with 0.25% glucose in a 96-well flat-based plate for 24 h at 37 °C. After incubation, the plates were washed thrice with phosphate-buffered saline (PBS). The attached cells were stained with crystal violet (1%) for 20 min. The dye bound to the clinging cells was dissolved in ethanol-acetone (80/20, *v*/*v*). The optical density of the solution was determined to be 570 nm [29]. The reference strain *A. baumannii* ATCC 17,978 was used as a positive control, while sterile TSB supplemented with glucose was used as a negative control.

The average reading of three repeated experiments was considered. Strains were separated into four groups: no biofilm producers, and poor, moderate, and strong biofilm producers based on the optical density (OD) produced by the *A. baumannii* isolates [30]. The mean OD of the negative control + three standard deviations (SD) was defined as ODc (cut-off OD). The degree of biofilm formation was reported as follows: strong biofilm formation (4 × ODc < OD), moderate biofilm formation (2 × ODc < OD < 4 × ODc), poor biofilm formation (ODc < OD< 2 × ODc), and no biofilm formation (OD < ODc) [30].

### 2.6. Molecular Identification and Characterization of A. baumannii Isolates

#### 2.6.1. DNA Extraction

*Acinetobacter baumannii* isolates were cultured in Luria-Bertani (LB) broth at 37 °C. DNA from fresh cultures of *A. baumannii* isolates was extracted using the QIAamp DNA Mini Kit (QIAGEN, Hilden, Germany). The quantity and DNA purity were assessed using a NanoDrop-1000 spectrophotometer (Thermo Fisher Scientific, Wilmington, NC, USA).

#### 2.6.2. Detection of Biofilm-Associated Genes by Polymerase Chain Reaction (PCR)

Detection of the *ompA*, *bap*, and *bla*_*PER*-*1*_ genes was performed using the primer sets, amplification conditions, and sizes of the detected amplicons as previously described by Badmasti et al. [5].

#### 2.6.3. Molecular Characterization of Antibiotic Resistance Genes

The *bla_OXA-51-like_* gene was used for molecular identification of *A. baumannii* [31]. OXA-type carbapenemases were detected using PCR. OXA-type carbapenemases include *bla_OXA-23-like_*, *bla_OXA-24-like_*, and *bla_OXA-58-like_* [31]. In addition, metallo-β-lactamase (MBL) genes, *bla_VIM_*, *bla_IMP_*, and *bla_NDM_* were assessed according to a previously studied protocol [32,33]. All primers were produced by Thermo Fisher Scientific Company, United Kingdom. Furthermore, the purified PCR products were sequenced by Macrogen, Inc. (Seoul, Korea) to retrieve the sequences of *bla_OXA-23-like_* and *bla_NDM_* genes that coexisted in one *A. baumannii* isolate. Sequence analysis was carried out using the NCBI internet-based BLAST tool, including the DDBJ/EMBL/GenBank database.

### 2.7. Repetitive Element Palindromic (REP)-PCR Genotyping

Repetitive element sequence-based PCR (REP-PCR) is a typing method that enables DNA fingerprinting to differentiate bacterial strains [34]. In the present study, we used REP-PCR genotyping of *A. baumannii* isolates recovered from ICU patients with pneumonia. REP-PCR was performed as described by Vila et al. [35]. Briefly, a 50 µL reaction mixture containing 100 ng of chromosomal DNA, 5 µL of 10 × Taq buffer, 1 µL of 10 mM dNTP mix, 1.5 U of Taq DNA polymerase (Promega, Madison, WI, USA), 50 pmol of each primer, and 2.5 μL of dimethyl sulfoxide were added [36]. The PCR conditions were as follows: an underlying denaturation at 95 °C for 3 min, followed by 30 cycles of denaturation at 90 °C for 30 s, annealing at 40 °C for 1 min, and extension at 65 °C for 8 min. The final extension step was carried out at 65 °C for 16 min. Lanes and bands were manually recognized against a 1 kb plus ladder lane (Jena Bioscience, Jena, Germany). Phylogenetic trees were designed using the PyElph version 1.4 [37]. Gel images of the fingerprint generated from REP-PCR of *A. baumannii* were analyzed using the gel documentation system UVitec (E4). Cluster homology of isolates was designed by Uviband map based on the unweighted pair group method using averages of the unweighted pair group method with arithmetic mean (UPGMA).

### 2.8. Galleria Mellonella Survival Assay

For in vivo examination of the killing effect of the biofilm-forming CRAB isolated from ICU patients with pneumonia, a *G. mellonella*-survival assay was performed. *G. mellonella* (greater wax moth) was supplied by the Pests and Plant Protection Department, National Research Center, Egypt. Under standard laboratory conditions, the experiments were carried out on the third generation of the rearing strain, which became more susceptible and healthy. Killing assay tests were carried out by injecting 10 µL of phosphate-buffered saline (PBS) suspensions containing 10^5^ colony-forming units (cfu) of bacteria/larva into the last right proleg, as previously described by Khalil et al. [38]. One group of larvae injected with 10 µL of PBS was set as a negative control to monitor for death caused by physical trauma. Another control group received no injection. The larvae were kept for 7 days and examined every 24 h for signs of death. Larvae that failed to react to physical stimuli or presented blackish discoloration of the larvae was reported as death. As high and low virulence reference strains, *A. baumannii* AB5075 and *A. baumannii* ATCC 19,606 were used, respectively. The experiments were performed three times, and the average reading was considered [39,40].

### 2.9. Statistical Data Analysis

Data analysis was performed using the Statistical Package for the Social Sciences software (SPSS version 17). Data were evaluated using the χ^2^ test or Fisher’s exact test. All statistical tests were two-sided. All analyses were performed using three separate experiments using GraphPad Prism software 6.01. The significance of differences was determined at *p* ≤ 0.05. Bivariate Pearson correlations between biofilm formation and pathogenicity of *CRAB* on *G. mellonella* were performed. The killing of *G. mellonella* by biofilm-forming carbapenem-resistant *A. baumannii* (CRAP) was analyzed using the Kaplan–Meier method. Log-rank test was performed.

## 3. Results

### 3.1. Bacterial Isolates

In this cohort study, two hundred and thirty (230) patients with HAP or VAP were included in the study. The male: female ratio was 1.7: 1 (146 men and 84 women). Among the collected samples, sputum was the most frequent sample (149), followed by ETA (51), BAL (22), pleural fluid (5), and bronchial wash (3). More than half of the clinical samples (60%, 138/230) showed significant growth. Single isolates were found in 111 samples and two organisms were found in 27 samples, while 92 samples showed either growth of non-pathogenic organisms or had no growth. A total of 124 isolates (124/165, 75.2%) were Gram-negative, while 35/165 isolates (21.2%) were Gram-positive cocci, and 6 isolates (3.6%) were *Candida* spp. *Acinetobacter baumannii* was one of the frequently isolated etiological agents in ICU patients with pneumonia (54/165, 32.7%) of the total isolates and (54/124, 43.5%) of the Gram-negative isolates. Other isolated Gram-negative pathogens included *Klebsiella pneumoniae* (29/124, 23.5%), *Pseudomonas aeruginosa* (36/124, 29%), and *E. coli* (5/124, 4%).

### 3.2. Antibiotic Susceptibility Testing and Prevalence of Carbapenemases Genes

All isolates exhibited full resistance to ceftazidime, and imipenem. Colistin had the greatest activity against *A. baumannii*, as all the isolates were susceptible to colistin. Fifty-two isolates (96.3%) were resistant to ampicillin–sulbactam and ciprofloxacin. Resistance to gentamicin, amikacin, and doxycycline was detected in 94.4%, 88.8%, and 90.7% of the isolates, respectively. The minimal inhibitory concentrations (MICs) of the isolates against the imipenem and colistin antibiotics were estimated using the broth dilution method. All isolates were imipenem resistant with MICs values ranging from 16–64 µg/mL (≥16 µg/mL). About 78% (42/54) of isolates had an MIC of 32 µg/mL against imipenem, while 18.5% of isolates had an MIC of 64 µg/mL. For colistin, all isolates were susceptible with an MIC of 0.25–2 µg/mL. Phenotypic inspection of carbapenemases activity revealed that 49 isolates (90.7%) were positive according to THT test.

PCR characterization of carbapenemase resistant genes was performed on all *A. baumannii* isolates from ICU patients with pneumonia. All isolates were *bla_OXA-51-like_* positive. *Bla_OXA-23-like_* was positive in 88.9% of the isolates (48/54). The *Bla_NDM_* gene was found in 27.7% of the isolates (15/54 isolates). *Bla_OXA-23-like_* and *bla_NDM_* genes coexisted in 25.9% (14/54) of the isolates. Conversely, none of these genes were detected in five isolates (9.3%). Additionally, none of the isolates were positive for *bla_OXA-24-like_*, *bla_OXA-58-like_*, *bla_IMP_*, or *bla_VIM_* genes (Table 1). Sequences of *the bla_OXA-23-like_* and *bla_NDM_*-1 genes that coexisted in one randomly selected isolate were analyzed in the DDBJ/EMBL/GenBank database and showed appropriate sequence homology to the corresponding genes.

### 3.3. Biofilm Assay and Prevalence of Biofilm-Associated Genes

Biofilm quantification showed that 61.1% (33/54) of the isolates were strong biofilm producers, while 27.7% (15/54) and 11.1% (6/54) showed moderate or weak biofilm production, respectively. PCR showed that all isolates were positive for *ompA*. *Bap* and *bla*_*PER*-*1*_ were identified in 14 (25.9%) and 6 (11.1%) isolates, respectively. *Bap* and *bla*_*PER*-*1*_ coexisted in 5.6% (3/54) of the isolates (Table 2). Among the *A. baumannii* isolates, the prevalence of *bap* was significantly higher in strong biofilm-producing strains (14/33, 42.4%) than in moderate (0%) and (0%) poor producers (*p* = 0.002). However, the distribution of *the bla_PER-1_* gene did not differ between the poor, moderate, and strong biofilm producers. The presence of *ompA*, *bap*, and *bla*_*PER*-*1*_ was significantly associated with strong biofilm producers in comparison to moderate and poor producers (*p* = 0.003) (Table 2).

### 3.4. Repetitive Element Palindromic (REP)-PCR Genotyping

**Figure 1:**
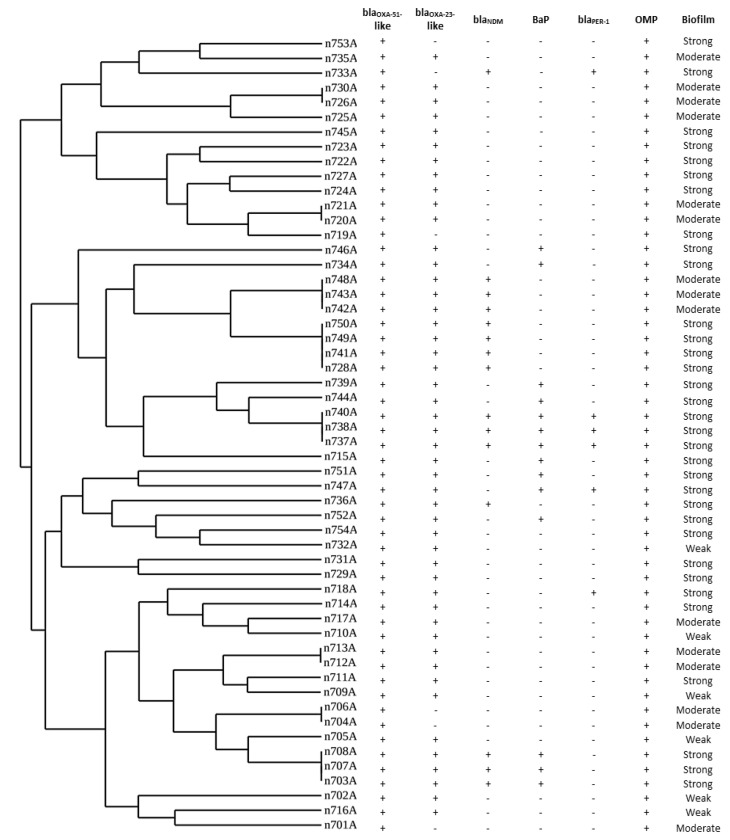
Dendrogram analysis of repetitive element palindromic (REP)-PCR genotyping of carbapenem-resistant *Acinetobacter baumannii* (CRAB) isolates. Clusters homology of isolates was designed by the Uviband map based on the unweighted pair group method using the arithmetic averages algorithm (UPGMA).

Four distinctive REP-PCR clusters (A–D) and two (746A, 715A) singleton isolates were derived from the band patterns. Thirteen *bla_NDM_* gene-carrying isolates were divided into four different identical patterns, while two isolates (733A and 736A) showed different unique REP-PCR patterns. The majority of *bla_NDM_*-carrying isolates belonged to group B. The same band pattern was found in half of the *bla_PER-1_* carrying isolates (3/6) (Figure 1).

### 3.5. Galleria Mellonella Survival Assay

**Figure 2:**
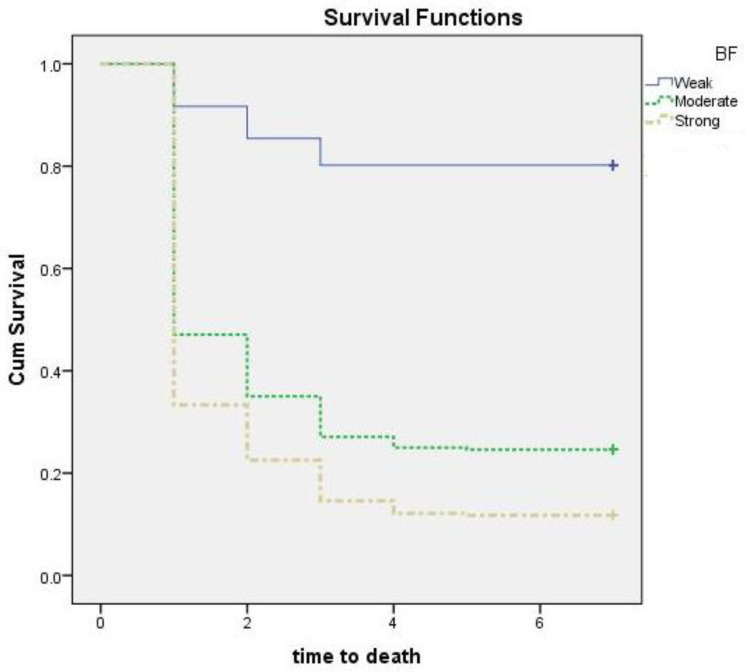
Pairwise comparison for the survival of *G. mellonella* larvae infected with clinical carbapenem-resistant *Acinetobacter baumannii* (CRAB); BF, biofilm. Isolates divided into three groups according to its biofilm-producing ability (poor (N = 6), moderate (N = 15), and strong (N = 33)). The larvae were injected with 10^5^ colony-forming units (CFU) into their last right proleg. Each isolate was tested against a group of 20 larvae, which were kept for 7 days at 37 °C, and examined for death every 24 h. The experiments were performed three times, and the average reading was considered.

In the biofilm assay, all isolates produced biofilms to varying degrees. Accordingly, we examined the susceptibility of *G. mellonella* to CRAB and analyzed the effect of the biofilm formation ability of the CRAB isolates as a killing hazard. After injection of larvae with approximately 10^7^ Cfu/mL, survival was observed daily for seven days, and a time-dependent survival assay was performed (Figure 2). No larva was missed during the assay, so censored lines are not present in the figure. Censoring is a type of missing data concern in which the time to event is not recorded (Figure 2). The survival time of *G. mellonella* treated with poor, moderate, and strong biofilm producing CRAB was (mean ± SD; 6 ± 2.1, 2.8 ± 2.5 and 2.1 ± 2 days, respectively). Survival in the negative control group was 100% (Table 3).

The difference in survival time between the group infected by the strong biofilm-producing CRAB and the other two groups (infected by the moderate and the poor biofilm producers) was statistically significant (*p* < 0.0001 for each comparison) (Table 3). In addition, *G. mellonella* survival was statistically different between moderate and poor biofilm producers (*p* < 0.0001). The cumulative survival of *G. mellonella* injected with CRAB with variable biofilm-production capabilities is shown in Figure 2.

After seven days, at the studied dose, seven isolates (13%) exhibited the ability to kill *G. mellonella* larvae, six of which were strong biofilm producers. Another seven isolates revealed a survival percentage of more than 50%, of which six were poor biofilm producers. Overall, 47/54 (87%) and 25/54 (46.3%) of the studied isolates were responsible for 50% and 90% mortality of *G. mellonella*, respectively (Figure 2). The Pearson correlation test was used to analyze the correlation between biofilm formation and *bap* and larval survival percentage. Biofilm formation and larval survival percentage showed a correlation coefficient value of −0.567, with *a p*-value < 0.001. In addition, *bap* and larval survival had a correlation coefficient of −0.457 (*p* = 0.001).

## 4. Discussion

The growing involvement of *A. baumannii* in serious clinical infections and their exceptional ability to develop antimicrobial resistance has become a critical concern [41]. The ability of *A. baumannii* to survive in the hospital environment and form biofilms increases its ability to cause different infections in patients. The respiratory tract is an important site of colonization and infection in ICU patients. *Acinetobacter* has been reported to colonize the nasopharynx, nares, or tracheostomy incisions. A prolonged stay in the ICU increases the possibility of colonization. Outbreaks of *Acinetobacter* infection, including pneumonia, have occurred in healthcare facilities worldwide [42]. In the present study, CRAB was the most common infectious agent responsible for pneumonia in the ICU, accounting for 32.7% of the cases. Various studies from across the world have also shown a high prevalence of *Acinetobacter* spp. in LRTIs (from 13% to 68%), which was higher than our findings [1,43,44,45].

Carbapenems are the antibiotics of choice for the treatment of infections caused by Gram-negative organisms resistant to third-generation cephalosporins. In the current study, carbapenem resistance was reported in all *A. baumannii* isolates from ICU patients with pneumonia, as has been observed worldwide [45]. Several methods were recommended for phenotypic characterization of carbapenemases in Enterobacteriaceae, including the Modified Hodge test (MHT), the Carbapenem Inactivation Method (CIM), and modified CIM [46]. However, these approaches revealed reduced efficiencies for the detection of *A. baumannii* carbapenemases due to the weak activity of OXA-type carbapenemases [47,48]. Pasteran and co-workers used Triton X-100 for the detection of NDM-type carbapenemases due to its potential to promote carbapenemase release. Many reports affirmed the improved sensitivity of THT for the detection of *A. baumannii* carbapenemase with a specificity of 100% [27,49]. In this study, THT was used to inspect carbapenemase activity instead of MHT. Although all the isolates were carbapenem resistant, 49 isolates (90.7%) showed positivity for the THT (positive carbapenemase activity). This could also be ascribed to mechanisms other than carbapenemase

In Gram-negative bacteria, such as *Acinetobacter* spp., carbapenem resistance is generally mediated by the production of carbapenemases enzymes. Carbapenemases can belong to class A (KPC 1 and 2), class B (VIM, IMP, NDM, and SIM types), and class D (OXA type) [50,51]. Class D carbapenemases include three subfamilies (*bla_OXA-23-like_*, *bla_OXA-24-like_*, and *bla_OXA-58-like_*) [52].

In our study, all isolates harbored *bla_OXA-51-like_* genes. Recognition of the intrinsic *bla_OXA-51-_*_like_ gene confirmed the identity of the *A. baumannii* isolates. The *bla_OXA-51-like_* gene has been reported to be a rapid and reliable tool for *A. baumannii* identification [24]. In the present study, *bla_OXA-23-like_* gene was the most frequently detected carbapenemase gene among the clinical CRAB isolates (88.9%), as previously reported [45,52,53], and the dissemination of CRAB carrying *bla_OXA-23-like_* was proven in many reports from Egypt [54,55,56].

The *bla_NDM_* gene was notably detected in comparison with other MBLs [45]. In the current study, 27.7% of the isolates harbored *bla_NDM_*. A study by El-Sayed-Ahmed et al. reported the widespread presence of *bla_NDM_* in Egypt [57], which has been recently identified worldwide [23,50,58]. The simultaneous presence of *bla_OXA-23-like_* with *bla_NDM_* genes in our CRAB isolates (25.9%) highlights the interplay and contribution of these genes to the resistance in our health care setting.

Moreover, none of the isolates in this study carried *bla_OXA-24-like_*, *bla_OXA-58-like_*, *bla_VIM_*, or *bla_IMP_*, which parallels the findings of Ghaith et al. [59]. Five isolates (9.3%) of *A. baumannii* in this study were positive for *bla_OXA51-like_* genes, and none of the other tested carbapenemase genes were detected. Carbapenem resistance in these isolates can be attributed to the increased expression of the *bla_OXA51-like_* genes provided by a strong promoter when associated with an ISAba1 7 bp upstream [60]. In addition, the contribution of other mechanisms of resistance to Carbapenem, such as altered permeability or other carbapenemase enzymes that were not tested in this study can explain the resistance of these strains to carbapenems. KPCs and GES-type (class A carbapenemases) were associated with carbapenem resistance in *A*. *baumannii* [8]. Lower drug affinity due to PBP downregulation [61], Efflux pumps (EP) mechanisms [62], and reduced membrane porin density [63] are examples of non-carbapenemase Carbapenem resistance mechanisms among *A. baumannii.*

Colistin is often the key component of many combination regimens used to treat MDR and CRAB infections. In the present study, all the CRAB isolates recovered from ICU patients with pneumonia were sensitive to Colistin. In Egypt, very few studies have reported colistin resistance among CRAB isolates from hospitalized patients [64]. However, colistin resistance has been previously reported [65,66,67]. Antimicrobial surveillance in hospitals is recommended for monitoring resistance to these drugs.

Biofilm development is one of the basic virulence traits of clinical *A. baumannii* isolates. Bacteria in biofilms exhibit a modified growth behavior that diminishes their susceptibility to different antimicrobial agents [68]. Along with the known conventional antimicrobial resistance mechanisms, various strategies contribute to the resistance of microorganisms in biofilms, such as slow or partial penetration of the antimicrobials into the biofilm, an abnormal microenvironment in the biofilm, and modified growth behavior of organisms within biofilms. These mechanisms are the outcomes of the multicellular nature of biofilms, which leads to antimicrobial resistance and failure of therapeutic interventions [69]. Approximately 61.1% of the *A. baumannii* isolates in this study were strong biofilm producers. Studies have reported that the survival time of biofilm-forming strains is much higher than that of those not forming biofilms (36 versus 15 days, respectively, *p* < 0.001) [70,71]. The ability of *A. baumannii* to develop biofilms would enhance its colonization and persistence, which facilitates the increased rates of nosocomial infections, especially device-associated infections [72].

Several variables, such as environmental circumstances and multiple cell signals, influence biofilm production by *A. baumannii* by affecting signaling, cell-to-cell interaction, and scaffolding roles [13,73]. In addition, various genetic determinants were correlated with surface adhesiveness and biofilm formation. These genes included *ompA, bap, and bla_PER-1_* [12]. Biofilm-associated genes provide a far-reaching view of surface adhesiveness and biofilm formation. In this study, we investigated the presence of *ompA*, *bap*, and *bla*_*PER*-*1*_ as biofilm encoding genes among CRAB isolates from ICU patients with pneumonia.

Our findings showed that *ompA* was present in all the isolates. In agreement, *ompA* was one of the most prevalent biofilm related genes among *A. baumannii* recovered from ICU patients, as reported by Zeighami et al. [74]. *OmpA* was assumed to be a part of many interactions with host cells, including attachment to epithelial cells and apoptosis induction. Thus, further studies of *ompA* regulation and expression will be essential to determine its effect on biofilm production among the studied strains [75,76]. *Bap* and *bla*_*PER*-*1*_ were detected in 25.9% and 11.1% of the isolates, respectively. After analyzing the association between biofilm production and biofilm-associated genes, *bap* and *bla*_*PER*-*1*_ were only detected among the strong biofilm strains. About 42.4% of strong biofilm isolates harbored the *bap* gene, while 18.2% were *bla*_*PER*-*1*_ positive. In one study that analyzed the genotype-phenotype correlation in biofilm-forming *A. baumannii*, the results revealed that the strains carrying *bap*, *bla*_*PER*-*1*_, and *ompA* genes tended to form stronger biofilms than the CRAB isolates lacking these genes. They also found that the *bap, bla_PER-1_,* and *ompA* genes were present in 81%, 39%, and 91% of the biofilm producers, respectively [77]. This was higher than what was observed in our study with regard to the *bap* and *bla*_*PER*-*1*_ genes, but similar to that observed with *ompA*.

The *bla*_*PER*-*1*_ gene is thought to be linked to biofilm formation by *A. baumannii* [76]. *A. baumannii* with *bla*_*PER*-*1*_ showed significantly higher biofilm production than those lacking the gene [16,78]. In the present study, *bla*_*PER*-*1*_ was present in 11% of the tested strains. However, a study by Bardbari et al. (2017) found no connection between biofilm production and the presence of *bla*_*PER*-*1*_ [79]. Thus, a possible explanation for this could be that *bla*_*PER*-*1*_ increases cell adhesion without necessarily affecting biofilm formation.

Recently, non-animal in vivo models such as *G. mellonella* have been used to characterize the virulence traits of different pathogens such as *A. baumannii*, *P. aeruginosa*, *Burkholderia cepacia*, *Bacillus cereus*, and several disease-causing fungi [19]. The ability of *G. mellonella* to be incubated at temperatures up to 37 °C makes it more appropriate for studying human pathogens [19]. Our study used *G. mellonella* to investigate the virulence of clinical CRAB isolates from ICU patients with pneumonia. The ability to kill of the strong biofilm-producing group was significantly higher than that of the moderate and poor biofilm producers (*p* < 0.0001 for each comparison). In addition, *G. mellonella* survival was statistically different between moderate and poor biofilm producers (*p* < 0.0001). These findings highlight the role of biofilm formation as a powerful virulence factor in CRAB. In accordance, a previous report of Li and co-workers used *Galleria mellonella* model to describe carbapenem-resistant hypervirulent *Acinetobacter baumannii* strains recovered from hospitalized patients [80]. In addition, the *Galleria* model was used to study the role of several factors in the fitness and pathogenicity of *A. baumannii* [81,82]. *G. mellonella* model was used to investigate the role of the surface antigen protein 1 (SurA1) gene in virulence and fitness of *A. baumannii* [81]. Moreover, Wand et al. reported that *A. baumannii* cells recovered from a biofilm produced higher killing rates than an equal number of planktonic cells [83].

PCR-based fingerprinting such as REP-PCR are simple, rapid, and low-cost methods with highly discriminatory power for discrimination between *A. baumannii*. An interesting review conducted by Sabat et al., concluded that the results of REP-PCR could be obtained in a relatively short period of time. This is also why this method is so inexpensive. REP-PCR is highly discriminatory for a wide range of bacterial species [84]. According to previous studies, the REP-PCR discrimination power was satisfactory and corresponded well with PFGE [85,86]. REP-PCR has been invaluable for the epidemiological investigation of hospital outbreaks. Many studies used PCR-based fingerprinting for the typing of clinical isolates of *A. baumannii* [74,86,87]. By typing of *A. baumannii* with REP-PCR, the obtained patterns were classified as four distinctive REP-PCR clusters (A–D) and two singleton isolates were retrieved from the band patterns. REP-PCR typing of clinical *A. baumannii* isolates by Meshkat et al. generated eight singleton isolates and ten individual clusters (referred to as A to J) [88]. REP-PCR was also utilized in an Iranian study by Zeighami and co-workers to type *A*. *baumannii* isolates from the ICU. Their REP-PCR findings reported 8 types (identified as A–H) and 21 subtypes. Within the subtypes, 7 singleton isolates and 14 different REP-PCR clusters were identified [74]. In our report, REP-PCR fingerprinting divided the isolates into four groups. Dominant patterns were detected among the 21 isolates that provided evidence of possible clonal expansion among different isolates.

## 5. Conclusions

The dissemination of strong biofilm-producing CRAB isolates among ICU patients with pneumonia is a challenging issue. Colistin remains the final treatment choice for controlling *A. baumannii* infections in Egypt. *Bap* and *bla*_*PER*-*1*_ were mainly associated with strong biofilm-producing *A. baumannii* isolates. As biofilm formation, *bap* gene expression, and *G. mellonella* killing had a strong positive correlation, the development of a new anti-biofilm approach could be a promising alternative for eradicating such pathogens. Surveillance programs should focus on monitoring strict hand hygiene compliance by all healthcare workers to prevent cross-contamination with these multidrug-resistant strains in resource-limited settings. Promoting audits and medical education can also support infection control programs.

## Figures and Tables

**Table 1:** Mechanism of carbapenem resistance among carbapenem resistant *Acinetobacter baumannii* (CRAB) isolates from ICU patients with pneumonia.

	*Acinetobacter baumannii* Isolates (N = 54) *			
Carbapenemase Genes	*bla_OXA-23-like_*	*bla_OXA-23-like_* + *bla_NDM_*	*bla_NDM_*	Only *bla_OXA-51_* Gene Was Detected
**N (%)**	48 (88.9%)	14 (25.9%)	1 (1.9%)	5 (9.2%)

* *Bla_OXA-51_* was detected in all isolates.

**Table 2:** Biofilm production (poor, moderate, and strong) among carbapenem resistant *Acinetobacter baumannii* isolates from ICU patients with pneumonia in relation to biofilm-associated genes (*ompA*, *bap*, and *bla*_*PER*-*1*_).

	Biofilm (*n* = 54) *						*p*-Value
	Poor (N = 6)		Moderate (N = 15)		Strong (N = 33)		
	N	%	N	%	N	%	
*Bap*							
Positive (N = 14)	0	0.0%	0	0.0%	14	42.4%	0.002 **
Negative (N = 40)	6	100.0%	15	100.0%	19	57.6%	
*bla*_*PER*-*1*_							
Positive (N = 6)	0	0.0%	0	0.0%	6	18.2%	0.117
Negative (N = 48)	6	100.0%	15	100.0%	27	81.8%	
Number of Genes							
1	6	100.0%	15	100.0%	16	48.5%	0.003 **
2	0	0.0%	0	0.0%	14	42.4%	
3	0	0.0%	0	0.0%	3	9.1%	

* All isolates were *ompA* + *ve.* ** Significant ≤ 0.05.

**Table 3:** Time to death (Survival) of *Galleria mellonella* injected by biofilm-producing carbapenem resistant *Acinetobacter baumannii* isolates from ICU patients with pneumonia.

	Survival (Time of Death)					*p*-Value
	Mean	SD	Median	IQR		
Poor	6	2.1	7	7	7	
Moderate	2.8	2.5	1	1	4.5	<0.0001 *^,a^
Strong	2.1	2	1	1	2	<0.0001 *^,b^ <0.0001 *^,c^

* Significant ≤ 0.05; SD, standard deviation; IQR, interquartile range; ^a^ moderate vs. poor, ^b^ strong vs. poor, ^c^ strong vs. moderate. Control larvae injected with 10 µL PBS (without *Acinetobacter* isolates) and the control group that received no injection exhibited 0% mortality.

## Data Availability

Not applicable.
